# Thinking about the action potential: the nerve signal as a window to the physical principles guiding neuronal excitability

**DOI:** 10.3389/fncel.2023.1232020

**Published:** 2023-08-28

**Authors:** Benjamin Drukarch, Micha M. M. Wilhelmus

**Affiliations:** Amsterdam UMC, Vrije Universiteit Amsterdam, Department of Anatomy and Neurosciences, Amsterdam Neuroscience, Amsterdam, Netherlands

**Keywords:** neuron, phase transition, axonal membrane-cytoskeleton, nerve impulse, signal transmission, communication

## Abstract

Ever since the work of Edgar Adrian, the neuronal action potential has been considered as an electric signal, modeled and interpreted using concepts and theories lent from electronic engineering. Accordingly, the electric action potential, as the prime manifestation of neuronal excitability, serving processing and reliable “long distance” communication of the information contained in the signal, was defined as a non-linear, self-propagating, regenerative, wave of electrical activity that travels along the surface of nerve cells. Thus, in the ground-breaking theory and mathematical model of Hodgkin and Huxley (HH), linking Nernst’s treatment of the electrochemistry of semi-permeable membranes to the physical laws of electricity and Kelvin’s cable theory, the electrical characteristics of the action potential are presented as the result of the depolarization-induced, voltage- and time-dependent opening and closure of ion channels in the membrane allowing the passive flow of charge, particularly in the form of Na^+^ and K^+^ -ions, into and out of the neuronal cytoplasm along the respective electrochemical ion gradient. In the model, which treats the membrane as a capacitor and ion channels as resistors, these changes in ionic conductance across the membrane cause a sudden and transient alteration of the transmembrane potential, i.e., the action potential, which is then carried forward and spreads over long(er) distances by means of both active and passive conduction dependent on local current flow by diffusion of Na^+^ ion in the neuronal cytoplasm. However, although highly successful in predicting and explaining many of the electric characteristics of the action potential, the HH model, nevertheless cannot accommodate the various non-electrical physical manifestations (mechanical, thermal and optical changes) that accompany action potential propagation, and for which there is ample experimental evidence. As such, the electrical conception of neuronal excitability appears to be incomplete and alternatives, aiming to improve, extend or even replace it, have been sought for. Commonly misunderstood as to their basic premises and the physical principles they are built on, and mistakenly perceived as a threat to the generally acknowledged explanatory power of the “classical” HH framework, these attempts to present a more complete picture of neuronal physiology, have met with fierce opposition from mainstream neuroscience and, as a consequence, currently remain underdeveloped and insufficiently tested. Here we present our perspective that this may be an unfortunate state of affairs as these different biophysics-informed approaches to incorporate also non-electrical signs of the action potential into the modeling and explanation of the nerve signal, in our view, are well suited to foster a new, more complete and better integrated understanding of the (multi)physical nature of neuronal excitability and signal transport and, hence, of neuronal function. In doing so, we will emphasize attempts to derive the different physical manifestations of the action potential from one common, macroscopic thermodynamics-based, framework treating the multiphysics of the nerve signal as the inevitable result of the collective material, i.e., physico-chemical, properties of the lipid bilayer neuronal membrane (in particular, the axolemma) and/or the so-called ectoplasm or membrane skeleton consisting of cytoskeletal protein polymers, in particular, actin fibrils. Potential consequences for our view of action potential physiology and role in neuronal function are identified and discussed.

## 1. Introduction: a short history and analysis of the development and popularity of the electricity-based conception of the action potential

“All animals that move have electricity in their bodies. Electricity is the only thing that’s fast enough to carry the messages that make us who we are. Our thoughts, our ability to move, see, dream, all of that is fundamentally driven and organized by electrical pulses. It’s almost like what happens in a computer but far more beautiful and complicated.”

(neuroscientist Rodolfo Llinas quoted in “Electricity’s spark of life” by Emily Sohn in Science News for Students, 29 September 2003).

The above quote, from pioneering cellular neurophysiologist Rodolfo Llinas, voiced in the early years of the current century, concisely highlights some commonly held beliefs by both modern day (neuro)scientists, students of neuroscience and lay-people about the central role of electricity in transporting the messages “that make us who we are.” This includes the claim that electricity is the only medium able to carry these messages at a sufficient speed to fulfill their task(s) which would be to drive, organize and integrate sensor-motor activity with brain and nervous system activity in humans (and other higher animals) in a way similar but not identical to binary electronic artifacts known as (digital) computers. In a broader sense, according to this notion, in order to optimize homeostasis and act as required, all animal species in possession of a nervous system will translate a large diversity of incoming physical and chemical signals from external- (e.g., light-rays and sound waves) as well as internal sources (e.g., local pH, pressure, temperature and chemical mediators like hormones/neurotransmitters or inflammatory stimulants) into electrical messages that travel along specialized cells known as neurons. In this model description, neurons, often as part of larger networks of similar yet structurally separate cells are instrumental in properly conveying the information contained in these messages between sensor and effector cells and for this purpose they are generally thought to use (bio)electricity as a medium (see for instance, Purves et al., 2012).

Much of this popular line of thinking about the fundamental properties and function of neurons and the physical, i.e., electrical, nature of the messages carried by them, in one form or another can be traced back to the seminal work on sensory and motor physiology of Adrian (1932) (and his predecessors and coworkers) in Cambridge, UK during the first three decades of the twentieth century. In fact, building on earlier insights gained from anatomy, (bio)physics and –chemistry, as well as physiology by the elaborate and ground breaking experimental and theoretical investigation of nerve physics and neuronal structure and wiring by scientists like, von Helmholtz, Du Bois-Reymond, Bernstein, Nernst, Lapicque, Hermann, Golgi and Cajal (and many others; see for recent historical overview, Drukarch et al., 2018 and references therein), and the clever use of technological innovations in signal amplification, in particular the thermionic vacuum tube (Garson, 2015), introduced during the first world war, the efforts of Adrian (1932) to characterize and understand the kind of signal(s) carried by diverse nerve fibers as well as the circumstances under which they occur and their role in nervous system physiology, have been instrumental in developing the modern “textbook” notions of the manner in which single neurons are functionally organized and act. Accordingly, in this we now know oversimplified picture (for more up to date descriptions see, e.g., Debanne, 2004; Bean, 2007; Kress and Mennerick, 2009; Debanne et al., 2013), individual neurons receive their input via a cluster of extensions called dendrites after which all incoming signals are processed and integrated in the cell body, also known as the soma. This processing and integration in the soma are done in such a way that the neuron either stays “silent” or “fires” a so-called action potential. Action potentials represent an abrupt, i.e., non-linear, change in the neuronal membrane potential which lasts up to a few milliseconds. Thus, in this scheme of events, which later was developed further to form the fundament of compartmental modeling of neuronal action in computational neuroscience(s) up to this day (see below), generation of action potentials is a “binary,” all-or-nothing, event during which either a single action potential with a standardized shape (and duration) is produced or nothing happens at all. Thereafter, following initiation in (or nearby, in the so-called axon hillock or axon initial segment) the soma, action potentials, as a “propagated disturbance” (Lucas, 1912; Rushton, 1937; Pareti, 2007), (will) propel forward along the surface of the axonal shaft, i.e., the neuron’s output channel, toward the synapse. At the synapse, finally, the message carried by the propagating action potential(s) will be communicated to coupled neurons (or effector cells), usually in the form of action potential-controlled release of chemical messenger molecules known as neurotransmitters. Thus, in a physical sense, action potentials, representing the “communicational currency” of the nervous system, are defined as self-propagating, regenerative, waves of electrical activity that travel along the surface of neurons (and other, so-called, excitable cells in both fungi, plants and animals which, however, unless indicated otherwise will not be considered here) (Patton and Thibodeau, 2015). It is this ability of neurons to generate and propagate action potentials which is usually referred to as neuronal excitability and that is often considered to be the principle physical characteristic of these cells, the specifics of which in terms of, for instance, shape, duration and velocity and accuracy of conduction of the action potential, are thought, amongst others, to determine timing, synchrony and overall efficacy of intra- and, in particular, inter-neuronal communication. In this spatio-temporal sketch of events allegedly underlying neural signaling, in particular the all- or none property of action potentials is thought to ensure the cellular transport of “encoded,” messages, i.e., information content, with high fidelity from the receiving end to the transmitting end of neurons (Bishop, 1956), thereby providing the basis for the concept, cum metaphor, that the nervous system (and especially the brain) operates as a digital counting mechanism with individual neurons acting “simply” as logic devices (Hameroff, 2022). In fact, based on then popular notions of information and communication and stimulated by the telegraph and telephone analogy used by many of his early twentieth century colleagues in neurophysiology to illustrate their view of the nervous system as a system of information transmission, it was Edgar Adrian who by the end of the 1920s was largely responsible for firmly rooting the notion of information in relation to descriptions of neuronal excitability by using words like “messages,” “signals” and “codes” in outlining the functional capacity of action potentials (Garson, 2003). Consequently, following the work of Adrian and his contemporaries, accurate characterization, mechanistic explanation and modeling of the neuronal action potential as an electrical phenomenon became a central focus of attention in experimental and theoretic neuroscientific research efforts and has remained so to this day.

Perhaps the most prized achievement of the above-described approach to characterization, explanation, modeling-and understanding of nervous system function in terms of generation, propagation, communication and organization of electrical pulses has been the development of a mathematical theory of signal processing in individual neurons (Keener and Sneyd, 2009). In the early 1950’s, building on earlier research in the lab of Adrian (1932), and using the newly introduced voltage-clamp technique to study nerve signals in squid giant axons, the work along this line culminated in the Nobel-prize winning theory and model of Hodgkin and Huxley (1952), which is considered by some as the “crown jewel” of (cellular) neuroscience and by others as “the first compartmental model” in computational neuroscience (Phillips et al., 2013; Almog and Korngreen, 2016). At the core of the HH-model, which describes the physiology of one cylindrical structure (Brette, 2015a), and, more in general, compartmental modeling of neurons lies the so-called cable equation which goes back to the seminal work of Lord Kelvin and others during the middle of the 19th century on signal decay in underwater telegraph cables (for recent historical review see Drukarch et al., 2018 and references therein). In the HH-model the propagating action potential, and therefore neuronal excitability, is described as a purely electrical phenomenon and the axon, along whose surface membrane the action potential moves, is (to be) modeled as a modified electronic circuit with the cell membrane, as “seat” of excitability, acting as a capacitor and the attendant “ion channels” as resistors (Phillips et al., 2013). Whilst the ion channels open and close in a time- and voltage-dependent manner, driven by their respective electrochemical gradient ionic currents (e.g., Na^+^, K^+^, CL^–^ and others) carrying charge passively flow across the axonal membrane generating a transient alteration of the transmembrane potential, i.e., the action potential, which is then carried forward and spreads over long(er) distances by means of both active and passive conduction (Purves et al., 2012). With the mathematical formulation of their theory HH could not only explain the results of dedicated experiments used to construct the model and fit the model parameters but also provide a remarkably accurate quantitative prediction of the shape, amplitude, threshold, velocity, refractory period and a number of other properties of the traveling electrical signal (Hodgkin and Huxley, 1952). As a consequence of this apparent descriptive as well as predictive power, the electronic circuit- and electrical/electrochemical conductance-based framework of neuronal excitability outlined in the HH theory and model was accepted fairly quick in broad areas of neuroscience, in general, and computational neuroscience and neurophysiology in particular. There it has served for the past 70 years, albeit often in some modified form, as a foundation for both theoretical and experimental research from molecular to circuit level and proved instrumental for building and maintaining the understanding of the primary function and *modus operandi* of the nervous system (including the brain) as a (binary) electronic information processing device with the attendant neuronal networks acting as an electrical wiring grid (Catterall et al., 2012).

Moreover, with their work HH also appeared to have provided unequivocal evidence to prove that the electrical phenomena associated with nerve excitation are a fundamental component of the nerve signal itself and not a sort of “epiphenomenon of an underlying, more essential process” (Piccolino, 1998). As formulated by Hodgkin (1964a) “the action potential is not just an electrical sign of the impulse, but is the causal agent in propagation.” In fact, to illustrate his line of thinking Hodgkin compared the conduction of action potentials to the burning of a fuse of gunpowder and explained that “the invariance of the action potential arises because the energy used in propagation does not come from stimulus but is released by the nerve fiber along its length” (Hodgkin, 1964b). Indeed, modern day neuroscientists, prompted by the acclaimed scientific success and popularity of the framework introduced in the HH theory and model, almost intuitively consider the presence of electrical current and voltage change as synonymous with generation and propagation of action potentials along neuronal extensions. From a historical perspective, however, it is important to realize that this has not always been the case. Originally, in reporting on investigations about the physical characteristics of what was called the “action current,” 19th century (bio)physicist/physiologist Emil du Bois-Reymond described the action potential in terms of electromotive force but he and his fellow biophysicists/-physiologists, both within Germany as well as abroad, despite intensive efforts (for recent review see, Drukarch et al., 2018), up to the mid-20th century had not been able to unequivocally identify the physical nature, i.e., the mechanism, of the relationship between this apparent electromotive nerve force and the action potential (see for example, Bowditch, 1886; Hardy, 1918; Bronk, 1933). More in particular, as noted for instance by Nigro (2020), following the conservation of energy discourse initiated by du Bois-Reymond’s colleague and friend, Herman von Helmholtz, to investigators involved, solving the outstanding question appeared in essence a matter of providing answers to issues like, where does the signal get its energy to propagate, how does it propagate, what is the fundamental, i.e., physico-chemical, nature of the signal and how does susceptibility to electrical stimulation of nerves relate to endogenous neuronal activity? Although aware of the fact that conductance of electric current accompanied the propagating nerve signal, lacking sufficient proof they were not prepared to definitively conclude if both phenomena were one and the same. Indeed, in his Nobel lecture, Adrian (1932), whilst contemplating the still unanswered question concerning the physical nature of the messages send by peripheral sense organs to the central nervous system, noted that “now it can be answered in much greater detail. It can be answered because of a recent improvement in electrical technique. The nerves do their work economically, without visible change and with the smallest expenditure of energy. The signals which they transmit can only be detected as changes of electrical potential and these changes are very small and of very brief duration. It is little wonder therefore that progress in this branch of physiology has always been governed by the progress of physical technique.” Thus, in emphasizing the crucial role of the sensitivity of electronic detection methods for the detection and study of neuronal signals in (neuro)physiology, Adrian did not actually answer the question concerning the physical nature of the relationship between the propagated signal itself and the accompanying electrical changes. It took the development of another, electrophysiological, technique, i.e., the voltage-clamp, which, following the general scientific acclaim for his and Huxley’s HH model, led Hodgkin to formulate his bold statement concerning the physical nature of the nerve signal (see above), and seemingly settle the issue. Good to note, however, is that the work of Hodgkin and Huxley had left unresolved some important questions concerning the mechanism(s) of the ionic permeability changes responsible for the action potential phenomenon. So, for example, at the time of introduction of the HH model, scientists had no established idea how the experimentally observed ionic currents between the interior of the nerve fiber and the extracellular space moved across the nerve membrane (Cole, 1975). In fact, the now well-established molecular entity generally known as “ion channel” was only a concept that designated the relative contribution of ions, in particular, Na^+^ and K^+^, to action potential generation and propagation rather than that it denoted an actual mechanism capable of explaining these phenomena. It took decades and development of yet another electrophysiological technique, the “patch-clamp” (Neher and Sakmann, 1976), to finally demonstrate that the opening and closure of voltage-sensitive macromolecular membrane-associated ion pores control and are responsible for ionic conductance through the membrane. Subsequently, the molecular identity and (atomic) structure of these ion channel proteins was elucidated using cloning techniques in combination with X-ray crystallography (Verkhratsky et al., 2006; Behrends, 2012). As a result of these discoveries, in the decades after its original formulation, the largely theoretic concept of “ion channel” in the original HH model slowly but surely emerged as a real molecular entity, apparently substantiating the fundaments and claims of the model and guaranteeing the continued popularity of the scientific framework it was built on. However, as it turned out, not everybody had been convinced.

## 2. The “electricity only” based conception of the nerve signal: facing criticism

As discussed in our recent historic overview of the topic (Drukarch et al., 2018), the contribution of the electricity centered conception of the propagating action potential, as formulated first by Hodgkin and Huxley and modified and extended later by others, to major advances in many areas of neuroscience cannot be overstated. Thus, at the molecular level, the HH model introduced a framework for the investigation of the structural and functional properties of ion channels, including the mechanisms of ion permeation, gating and selectivity. At the cellular level, the model proved very useful in predicting state variables that control the induction and time-course of action potentials, such as threshold and refractory periods, whilst at the circuit level it was instrumental in helping computational neuroscientists to understand and model brain function in terms of neuronal integration and circuit level information processing (Catterall et al., 2012). As such, the HH model has repeatedly and justifiably been highlighted as “the most important model in all of the physiological literature” (Keener and Sneyd, 2009).

However, despite its acclaimed success, over time, on numerous occasions and for various reasons some of the basic tenets and claims on which the validity of the electricity-based conception of the action potential rests have been called into question. Thus, in recent years, for example, from within computational neuroscience and neurophysiology a debate picked up concerning the status of the “neural coding” metaphor, introduced originally by Adrian (1932) to describe the relationship between stimulus intensity (i.e., energy input) and rate of action potential firing in nerves (i.e., consumption of energy input; see above), as an apt and helpful tool to characterize and measure the information content carried by action potentials about the objective properties of external stimuli (for excellent reviews see, Brette, 2015b,^2019^). Related to this, the mainstream “HH framework” has also been criticized for its treatment of neurons as “essentially inanimate objects,” i.e., threshold logic devices, in which information processing is considered solely in terms of membrane and synaptic activities whilst ignoring other, (intra)neuronal, biological variables (Hameroff, 2022). This apparent lack of “biophysical realism” in today’s, HH-based, modeling, understanding and interpretation of neuronal activity is also noted as a serious shortcoming by Almog and Korngreen (2016), who, in referring to De Schutter (2008), observe that “the Hodgkin and Huxley’s model is a purely electrical one. It makes no reference to biochemistry, changes to cytoplasmic ionic concentrations, or other intracellular processes. Even today, compartmental models remain electro-centric, a legacy partly responsible for the lamentable lack of common language between computational neuroscience and chemo-centric systems biology.” Indeed, already shortly after introduction of the HH model, the change in membrane potential accompanying action potential generation was described as “a conveniently recorded sign of the cell’s excitation, although the metabolic and electrochemical events back of the depolarization may be a more essential activity, of which the electrical phenomena are a consequence” (Bishop, 1956). This criticism, which centers around the perceived lack of consideration in the HH electricity-based framework of computational neuroscience and neurophysiology of other, perhaps even more “proximal,” neuronal events and its consequent inability to account for and integrate these into a comprehensive view of neuronal excitability appears to reflect a return to the old(er) discussion on the physical nature of the “nerve force” initiated by Bowditch (1886) at the end of the 19th century and thereafter repeatedly picked up by others but largely ignored in mainstream neuroscience (Drukarch et al., 2018). In an attempt to explain this, in his view, unwelcome state of affairs, Ichii Tasaki, one of the most vocal opponents of the modern, prevailing view of neuronal excitability, observed that “with the advent of the age of electronic engineering, …., the traditionally close tie between physical chemistry and physiology was weakened considerably. Driven by the increasing need for advanced knowledge of various electronic devices employed in their experiments, investigators of physiology started to interpret physiological findings in terms of electronic engineers’ concepts, e.g., positive feedback, channels, gates, equivalent circuits, and less emphasis was placed, ……, on physicochemical approaches” (Tasaki, 1982). In recent years, however, inspired by the large body of work of Tasaki and others from the 1970’s onward, the interest in developing such a broad(er), physico-chemical framework of neuronal excitability, has been rekindled again. Apart from theoretical considerations outlined above, this renewed interest was stimulated also by the reporting of a variety of non-electrical physical manifestations of the action potential (mechanical, thermal and optical) that were found to (co-)occur and move in synchrony with the propagating nerve signal but which cannot be accommodated (at least not in a straightforward way) within the purely electrical view, i.e., changes in charge and electrical potential caused by a bistable switch in membrane conductivity of Na^+^ and K^+^ ions, as presented in the HH formalism (Andersen et al., 2009; Mueller and Tyler, 2014). Within this context, particular attention was paid to the heat release and absorption and swelling and subsequent contraction of the axon largely coinciding with the depolarization and repolarization phase of the action potential, respectively, and associated with a rise and fall in intracellular pressure (for recent overview see, Drukarch et al., 2018; for additional interesting notions concerning the relationship between axonal activity-dependent swelling and the release of neuroactive agents, see Fields and Ni, 2010; Fields, 2011). As acknowledged by Hodgkin (1964b), being dissipative in nature, the HH model fails to provide a plausible explanation for these (at least partly) reversible, non-electrical manifestations of the nerve signal and which thereby provided the impetus for attempts to improve, extend or even replace it with a more comprehensive view. Together, as a group these activities shared their interest in answering the three outstanding questions originally formulated by Terakawa (1985) about the mechanical responses he had observed during axonal excitation: (1) which cellular component(s) do they arise from?, (2) how are they produced?, and (3) what is their physiological significance? For this reason, amongst others, changes in membrane capacitance resulting from electric potential driven variation in membrane thickness, and the possible involvement of neuronal structures for which there is no clearly defined place in the purely electrical conception of the action potential, like the lipid bilayer membrane and/or the neuronal cytoskeleton, became the focus of attention and were considered in varying forms and to different degrees (for critical reviews see, Mueller and Tyler, 2014; Drukarch et al., 2018, 2022; Holland et al., 2019; Jerusalem et al., 2019). Whilst agreeing that, contrary to the mainstream view, the nerve signal is to be more properly understood as an electro-mechanical or even multi-physics wave, in order to prevent unnecessary misunderstandings, it is vital to note that in these attempts to explain and model the experimentally observed electro-mechanical couplings along the axon two fundamentally different theoretical approaches to (bio)physical modeling were used, i.e., a bottom-up vs. a top-down one. Using the bottom-up, i.e., mechanistic and thus synthetic, approach, in order to explain (at least some) of the unaccounted-for observations, functional involvement of specific forms of mechanical signaling in neuronal excitability, like electrostriction and (reverse) flexoelectricity, was proposed (El Hady and Mehta, 2015; Chen et al., 2019). In the described models, the mechanical manifestations of the nerve signal are presented as epiphenomena either inevitably accompanying the electric manifestations or resulting from the mechanism underlying the electric action potential. Thus, as described by Jerusalem et al. (2019), the various models using the bottom-up approach, “similar to the coupling of Nernst’s theory of the electrochemistry of semi-permeable membranes to the laws of electricity (e.g., Ohm’s and Coulomb’s laws) and Kelvin’s cable theory in the purely electrical HH model of the nerve impulse, aim primarily to itemize the different physics involved in the experimental observations linking them by physical laws.” As a consequence, however, of their focus on the physics of interest for a particular purpose or application (for instance, in the HH model that is explanation of the electrical manifestations of the action potential), they suffer from a lack of generalization. This potential, albeit aim- and purpose-dependent, drawback is overcome using the top-down method in which the different physical manifestations of the traveling nerve signal are considered as intertwined forms of energy. Thus, in order to fulfill their ultimate objective, that is unification of all (known) physical manifestations of the nerve signal, top-down modeling efforts, being of a non-mechanistic, analytic and primarily holistic, nature, have focused on determining the framework that best addresses all aspects (electrical, mechanical, thermal, etc.) of this wave phenomenon at once (for more extensive discussion see, Drukarch et al., 2022). In doing so, in contrast for example to the prevailing electrical conception of neuronal excitability, they have largely abstracted away from molecular-level entities, like ion channel proteins, and instead put an emphasis on the importance of the collective, physico-chemical, material properties and/or macroscopic thermodynamics of the axonal membrane and axonal cytoskeleton. Almost inevitably, therefore, in these efforts to develop a unified top-down modeling framework of the nerve signal as an intertwined multi-physical wave phenomenon, a so-called phenomenological description of the system of interest (axonal membrane, for instance) was used in which the behavior of the system is described solely in terms of “coarse-grained” variables without reference to the microscopic details of the system (Tasaki, 1999a and references therein). Using this approach, the abrupt (and at least partly reversible) multiphysical (i.e., electric, mechanical, thermal, etc.) changes coinciding with axonal excitation were interpreted and modeled as manifestations of sudden structural transformations, or phase transitions, in the axonal membrane and/or intimately associated parts of the cytoskeleton (Fernandes de Lima and Hanke, 2016). Within this context, phase transitions, most simply defined as a shift of a system from one identifiable state of order to another elicited by, often subtle, changes in a so-called control parameter (Heffern et al., 2021), offer an especially attractive explanatory concept as they are well known to be accompanied by drastic changes in the material properties of the system of interest (including changes in compressibility, conductance and heat storage), are induced by relatively small variations in external conditions (like, electrical field, temperature, pH, calcium ions) and, similar to action potentials, can appear as on/off switches (Kuklin et al., 2020; Heimburg, 2023).

Arguably, the most extensively discussed of the novel conceptions of the action potential as an electro-mechanical wave phenomenon is the highly controversial “soliton model” of Heimburg and Jackson (2005). Building on earlier theoretical work by Kaufmann (1989) on the macroscopic thermodynamics of lipid membranes and the propagation of acoustic pulses in membrane interfaces, in this, often poorly understood (for extensive treatment of the issues involved see, Drukarch et al., 2022), attempt to reconcile all of the established physical, electrical as well as non-electrical, manifestations of the propagating action potential in one physico-chemical framework, HJ derived a wave equation for single electromechanical pulses in lipid membranes (a soliton) and proposed that the quantized, all-or-none, conduction events coupled with reversible mechanical (e.g., thickness, swelling, pressure) as well as thermal changes observed during action potential propagation can be explained by considering the nerve signal as an “ acoustic pulse along the membrane” in which the movement of a single adiabatic wave (a soliton) through the lipid bilayer is responsible for axonal conduction of the pulse. As recently discussed by us (Drukarch et al., 2022), in their work HJ emphasize the importance of non-linear state changes in the lipid membrane during propagation of the pulse. In particular, they stress that the pulse-like shape of the soliton is the consequence of the non-linear nature of membrane compressibility near to phase transition in the membrane. In the words of HJ, “during the pulse, the membrane is partially moved through a phase transition from a liquid-disordered membrane state to a solid-ordered state” (Jackson and Heimburg, 2020). This lipid phase transition is accompanied by a density change which, as the direct consequence of increased membrane tension (measured as change in lateral membrane pressure) inevitably results in a change in membrane area and thickness, change in membrane charge density and, therefore, membrane potential. Because of the (at least partly) reversible nature of the structural changes in the membrane (i.e., compression followed by relaxation measured as a change in density of the lipid molecules), once moving, the self-sustaining and localized density pulse will present itself also as a voltage pulse, generally known as the propagating action potential in the electric HH framework. Thus, in this thermodynamics-based framework, movement of the action potential relies on the same fundamental principles that cause the propagation of sound waves in a material instead of the flow of ions or current. Accordingly, the electro-mechanical phenomenology of the nerve signal emerges naturally from the collective properties of the axonal membrane, in which a compression wave propagates, analogous to a sound wave. In support of the validity of the assumptions underlying their model, HJ note that in fact “the associated changes in the thickness of the membrane, the length of the axon and the reversible release of the latent heat have all been found experimentally “(Jackson and Heimburg, 2020). Nevertheless, it should be acknowledged that in its present form, although being able to account in a qualitative sense for a number of aspects of neuronal excitation not covered before, the HJ soliton theory thus far falls short of doing so in a quantitative manner. In this respect, from a modeling perspective it still lacks compared to the HH model which does provide a quantitative account of both action potential generation as well as propagation, albeit covering only the electric characteristics of the nerve signal.

More important, the HJ soliton theory has faced strong criticism and met fierce opposition from mainstream neuroscience for its apparent inability to capture the well-known annihilation phenomenon occurring when two action potentials, running along the same nerve fiber from opposite directions, run into each other (Follmann et al., 2015; Berg et al., 2017). In fact, studied and reported on for the first time by Tasaki (1949) and predicted by the HH model as the consequence of the inactivation of Na^+^ -channels during the so-called refractory period, in contrast the HJ theory predicts that, similar to sound waves in air, such colliding nerve pulses will penetrate each other and continue unaltered. Despite some experimental evidence provided by HJ and coworkers to sustain their claim (Gonzalez-Perez et al., 2014, 2016), this issue, however, remains highly contentious and is eagerly used to argue against the scientific validity of the HJ soliton theory as such and, more in general, the thermodynamic foundations it is built on (see for instance, Follmann et al., 2015). However, as recently pointed out by us (Drukarch et al., 2022), this is not correct as the HJ soliton “model” in its current form should be properly understood as “the “simplest” (first order non-linearity and dispersion) description that tries (and to a certain extent succeeds) to capture most but not all of the essential features of an adiabatically propagating phase transition in a membrane. In fact, it is the first attempt at a quantitative description of this phenomenon based on thermodynamics and it should be considered as such.” Indeed, incorporation of higher order terms into the soliton “model” has been shown to be sufficient to meet the criticism and demonstrate that the thermodynamic theory does allow for annihilation of colliding action potentials both mathematically (Mussel and Schneider, 2019), as well as experimentally (Fillafer et al., 2017; Shrivastava et al., 2018).

In a similar vein, another bone of contention between representatives of the mainstream, majority view of the action potential as an electric pulse, and the small minority interpretation of the action potential as a multiphysical, thermodynamic wave running in the membrane, is being addressed. This concerns the *a priori* argument that lipid membrane phase transitions may be a sort of, biologically irrelevant, experimental artifact apparently detectable under specific, i.e., artificial, conditions in “pure” lipid layers, but unlikely to occur in living cells under physiological conditions. Whilst pointing to the potential danger to cellular viability, allegedly caused by an increase in membrane permeability resulting from formation of temporary membrane pores, and/or considerable changes in membrane pressure accompanying lipid phase transitions (Meissner, 2018), earlier arguments about the potential importance of phase transitions in biology are ignored (Changeux et al., 1967; Trauble and Eibl, 1974; Melchior and Steim, 1976). Moreover, in our opinion, following this line of reasoning the importance of recognition of the link between the expected physical phenomenology of these perceived “dangers to cellular viability” and the experimentally supported understanding of the traveling nerve signal as an electro-mechanical wave (encompassing both electrical, mechanical and other manifestations) is overseen. Therefore, in light of these theoretical considerations it is important to note that, similar to observations made in artificial lipid membrane models (Kang and Schneider, 2020), in recent years some preliminary experimental data has been provided to show that as the action potential moves forward, in line with Kaufmann’s thermodynamic theory, the cell membrane of excitable plant cells sequentially condenses (freezes) and melts (relaxes-rarefaction) during the depolarization and repolarization phase, respectively (Fabiunke et al., 2020). In addition, experimental evidence has been presented demonstrating sharp, localized and reversible phase transitions in the surface membranes of cultured neuronal cells triggered by temperature changes and modified by pH (Fedosejevs and Schneider, 2022). Although in need of independent verification and further experimental elaboration, pointing to a significant change in thermodynamic state of the membrane as expected during (reversible) phase transition, these data provide support for the central idea upon which Kaufmann’s and HJ’s thermodynamic theory of the nerve signal is built which is that the wave front of the action potential propagates as the result of a reversible- elastic- process similar to the propagation of sound and not as the outcome of an irreversible- diffusive- process alike the “burning of a fuse of gunpowder,” as proposed by Hodgkin for the HH model (Hodgkin, 1964b). At the same time it is good to acknowledge that, for the purpose of “realistic” explanation and modeling of the multiphysical characteristics of propagating action potentials in live neurons, within the thermodynamics- and mechanical framework supported by HJ and others more complexity might have to be ruled in because the elastic and viscous properties of (artificial) lipid bilayers, which together control the propagation or attenuation of mechanical signals in cell membranes, have been shown, for instance, to be strongly regulated by membrane cholesterol content (Bennett et al., 2009; Al-Rekabi and Contera, 2018). For this reason, it is important to note that cholesterol is not usually included as part of the phospholipid layers generally used by membrane biophysicists to study and model the physical characteristics of cell membranes. Moreover, also other physico-chemical properties of the axon itself, in particular those of the (sub)membranous axonal cytoskeleton, play a crucial role in determining phase transition and other biophysical characteristics of the membrane and will therefore have to be taken into account (for recent review see, Jerusalem et al., 2019 and references therein). In fact, as emphasized before by Tasaki and coworkers and discussed in the next paragraph, for this purpose another form of structural transformation (i.e., phase transition) might have to be considered as a vital and causal (co)determinant of the particulars of axonal excitability.

## 3. The (sub)membranous axonal cytoskeleton and the physico-chemical basis of neuronal excitability

Albeit offering an incompatible reading of the proffered physics [discussed by us in Holland et al. (2019)], in both the electricity-centered HH-model as well as the thermodynamics and acoustic physics-based description of the nerve signal as presented by HJ and similar-minded (membrane) biophysicists, the axonal membrane and/or attendant voltage-gated ion channels as such are conceived as the “seat” of excitability. Indeed, in an attempt to answer his questions concerning the physical nature and (sub)cellular origin of the mechanical changes observed during axonal excitation, Terakawa (1985) put forward that the pressure response is likely to originate in the so-called axolemma (i.e., cell membrane surrounding the axon) and noted that depolarization presumably causes electrostriction [i.e., mechanical deformation of a dielectric (insulator) in the presence of an electric field] underlying the changes in membrane thickness and tension. Trying to align his ideas with the majority opinion on the physical, i.e., electrical, nature of axonal excitability, in musing about the potential consequences of his findings for neuronal physiology, Terakawa (1985) moreover hypothesized that changes in mechanical lipid membrane forces, in particular membrane thickness, may affect the gating mechanisms of ion channel proteins, an idea since then strongly substantiated by experimental data where it is sometimes described as the “force from lipid” (Anishkin et al., 2014). Thus, irrespective of the explanatory framework applied, as an extension of the mechanistic HH model of the action potential or in the form of its best-developed non-mechanistic competitor, only the physico-chemical characteristics of the axonal membrane treated as a somewhat decoupled interface have been considered in explaining the physical underpinnings of neuronal excitability in general, and the electrical and non-electrical manifestations of the propagating action potential, in particular.

However, although usually perceived to function primarily in providing mechanical and structural support to individual cells, including neurons, over the years there has been ongoing speculation about the involvement of the fibrillar components of the cytoskeleton in specific cellular signaling events at the cell surface. In fact, as part of the efforts to understand their structure-function relationships, the key elements of the (nano)fibrous cytoskeleton, i.e., actin-based microfilaments, intermediate- or neurofilaments and microtubules (Kevenaar and Hoogenraad, 2015), have been shown to be connected through the cell membrane to the fibrous protein-based building blocks of the extracellular matrix (Schwarz and Gardel, 2012). As such, cellular assemblies in tissues, organs and even the whole body are sometimes considered to represent a continuous fibrous communication network in which the cytoskeletal fibrillar elements serve to connect cell nuclei (and other subcellular organelles) to the materials of the extracellular matrix and function to integrate (intra)cellular signaling in acting as the biological analogues of wires (for reviews see, Friesen et al., 2015; Barvitenko et al., 2018). In this context it is of special interest, that from the 1970’s onward it has been widely recognized that a complex and highly specialized macromolecular network, consisting mainly of aligned actin microfilaments, is in close physical contact with the axolemma, being located directly under and, at least according to older literature (see below for further discussion), running parallel to the lipid bilayer along the long axis of the axon (Metuzals and Tasaki, 1978). Being similar in its general principles of structural organization to its counterparts in other cells, the surface of the axon may therefore be considered to comprise of both the axolemma, a lipid bilayer membrane with transmembrane and membrane-bound proteins and sugars, and an underlying, primarily actin-based, cortical cytoskeleton or cell cortex that is connected to the membrane by specific and non-specific molecular interactions (Sitarska and Diz-Munoz, 2020). In the older literature, this cell cortex is also referred to as the ectoplasm which led some investigators to describe the axonal surface as the axolemma-ectoplasm complex (Metuzals and Tasaki, 1978; Matsumoto, 1984). Although its existence is acknowledged in recent papers and modern textbooks on the topic, in line with commonly held scientific intuitions investigation and discussion of its importance in determining neuronal structure and function is usually restricted to consideration of its contribution to transport of materials (e.g., membrane and secretory proteins) and its mechanical role in dictating neuronal shape, growth and movement. In fact, studied most extensively for the actin cortex of animal cells, today most biological membranes are thought to be mechanically stabilized by a cytoskeletal structure that provides not only mechanical rigidity but also exerts forces on the membrane (Turlier and Betz, 2019).

In comparison to this very active field of biophysical investigation, however, only limited attention has been paid to the possibility that the cortical, actin-based, cytoskeleton of axons might participate (also) in neuronal excitability, serving the purpose of nerve signal generation and/or conduction. Evidence shows that from a polymer physics point of view the actin cytoskeleton can be considered as a polymer gel (Joanny and Prost, 2009), which displays the physico-chemical properties of a cation exchanger. This indicates that its protein parts are rich in anionic amino acids, for instance, glutamic and aspartic acid, which are considerably ionized at physiological pH. Under resting conditions, this cortical cytoskeleton or ectoplasm forms a dense polymer-gel matrix which is cross-linked by Ca^2+^ ions and so-called structured water. The “structured” water content of this superficial gel layer of the axon is governed by the balance between two opposing forces, namely, the attractive force exerted by Ca^2+^ upon the negatively charged sites on the polymer strands (which tends to make the gel shrink) and the osmotic pressure exerted by the unbound cations in the gel (which tends to expand the gel). Using an impressive array of axonal preparations from animal sources in combination with a variety of experimental conditions and biophysical measurement techniques, the predicted connection between the physico-chemical state of the “axolemma-ectoplasm complex” and action potential generation and propagation was probed by a broad group of neuroscientists, including perhaps most famously Ichii Tasaki. Moreover, in order to further elaborate on important aspects of his ideas about the role of the macromolecular cortical cytoskeleton in neuronal transmission, Tasaki coupled his experiments on axons with studies on synthetic, polyanionic gels. This latter set of experiment, with spheres or thick fibers of cross-linked (poly)sodium acrylate, importantly showed volume shrinkage of the Na^+^-form on titration with CaCl_2_, in which the transition sharpened in the presence of a bathing solution of appropriate concentration suggesting a cooperative transition at a critical Ca^2+^ concentration (for overview and discussion of this large set of data see, Tasaki, 1999a,b, 2008 and references therein).

**FIGURE 1:**
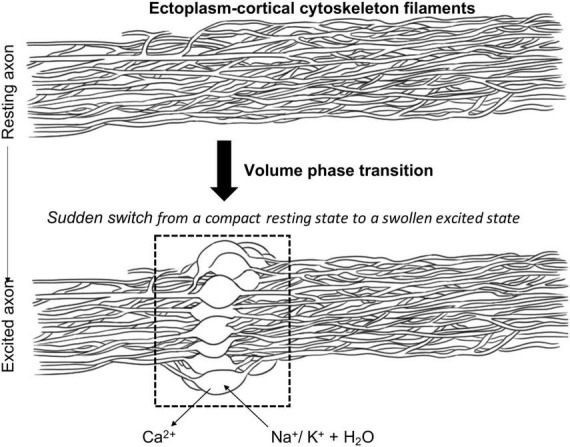
Proposed mechanism of volume phase transition in the ectoplasm-cortical cytoskeleton filaments as a consequence of axonal excitation. Adapted from Wnek (2016).

Together, the results of their investigations led Tasaki and colleagues to propose that, during axonal excitation, influx of Na^+^ ions (accompanied by “free” water molecules) displaces Ca^2+^ from the gel layer near the axolemma, thereby disrupting calcium bridges between the negatively charged protein polymer chains (Figure 1). Thus, as a reflection of the physico-chemical nature of the excitation process, the ectoplasm supposedly undergoes a reversible and abrupt structural change when monovalent cations, e.g., Na^+^, are substituted for the bivalent counter ions (i.e., Ca^2+^) (Figure 1). This brings about a sudden rise in the water content of the gel matrix resulting in a large enhancement of cation mobilities accompanied, in its turn, by a shift of ion-selectivity in favor of hydrophilic cations like Na^+^. Overall, these cooperative events cause the ectoplasmic gel matrix to loosen and expand due to an increase in the repulsive electrostatic interactions (Tasaki, 1999a,b, 2008). In this view then, which is largely in line with the more than a century- old, but long-forgotten, proposals of Loeb concerning the role of ion-exchange chemistry in managing excitability in living tissues (Loeb, 1906), the colloidal state of the ectoplasm gel polymer is regulated tightly by the ratio of the concentration of bivalent Ca^2+^ ions to that of monovalent, in particular Na^+^ but also K^+^, ions. Accordingly, trusting that the ionic microenvironment allows for facile switching between the two states, the processes of neuronal excitation and conduction are considered as manifestations of a sudden, discontinuous, i.e., first-order, volume phase transition in the superficial macromolecular cytoskeletal gel layer of neuronal axons characterized by a “rapid change from a compact, Ca^2+^-rich, resting state to a swollen, calcium-deficient, excited state” (Tasaki, 1982). Consequently, as originally formulated by Williams (1970), propagation of an action potential is viewed as the electrical manifestation of a “running wave of structural changes along the membrane” produced by diffusion and binding of calcium ions (Iwasa and Tasaki, 1982). By emphasizing the significance of the rapid (but reversible) switching due to a cooperative but discontinuous transformation between the compact and swollen states, respectively, Tasaki moreover argued that this structural change in the cortical gel layer of axons, in the form of a propagating volume phase transition, is likely not only to participate in the voltage transition during excitation (for a more detailed treatment of the physical chemistry involved in this line of reasoning, see, Wnek, 2016; Wnek et al., 2022 and references therein), but is also responsible for the (reversible) swelling of the axon and temperature rise well known to accompany the electrical signs of the nerve signal. As such, Tasaki claimed that, in fact, it is the tuning of the bistable state of the ectoplasmic gel, that is the continuum of states, sometimes referred to as “interphase,” ranging from the Ca^2+^-contracted to the swollen Na^+^/K^–^ form, respectively, that ultimately determines neuronal excitability (Tasaki, 1999a,b, 2008). In this view, excitation (electrical, mechanical, thermal or otherwise) destabilizes the resting, compact-form, leading to a fast exchange with monovalent ions, in particular Na^+^. This would turn a “resting” zone of the cortical cytoskeletal gel layer into an “excited” zone which can move (in principle, not only in an anterograde but also retrograde direction) creating a new “excited” zone whilst the previous “excited” zone relaxes back to its “resting” state. As part of this relaxation process the water molecules surrounding the Na^+^ -bound form are released upon Ca^2+^-binding, during which two moles of Na^+^ are exchanged per mole of Ca^2+^ bound. Furthermore, although requiring experimental verification, the proposed mechanism underlying the process of ectoplasmic relaxation might also provide a physico-chemical explanation for another phenomenon typically associated with neuronal excitation. Thus, Wnek (2016) speculates that “Conversion back to the Ca^2+^-compacted resting state may not be immediately reversible, with instead ion exchange leading to a more compact (more so than the original resting state) transient state that over a short time relaxes to the equilibrium resting state as the result of the kinetics of multistep ion-exchange processes and water redistribution around mobile ions and fixed charges. Such a relaxation process may contribute to the well-known refractory period where some time must pass before an impulse can be triggered once again.”

Using the ideas and results of the above-described work of Tasaki and others, Wnek (2016), moreover proposed that, from a spatial-temporal perspective, it is to be expected that the cytoskeletal lower resistance, excitation zone, “advances axially via a series of closely coupled ion exchange events that occur radially.” More in particular, he suggests that these coupled ion exchange events would be controlled by the presence of ion channels and pumps in the axolemma that “could ensure that the [monovalent]/[divalent] balance is set correctly for a hair-trigger phase transition between resting and excited states.” In this context, it is therefore intriguing to note that Na^+^ -ion channels have been reported to be distributed in axons in a periodic pattern that closely correlates with cytoskeletal rings of actin and spectrin (Xu et al., 2013), indicative of a direct functional and perhaps even structural interaction between these cytoskeletal filaments and supposedly membrane-anchored ion channel proteins. Furthermore, strong experimental support for the importance of the axonal ectoplasmic network in action potential generation and propagation has been drawn from the results of studies in which the so-called axoplasm (the cytosolic gel inside the axon) was removed and replaced by an appropriate salt solution with a Ca^2+^ -containing medium available on the outside. Summarized by Wnek (2016), key observations from this large body of work show that (1) the axoplasm gel is apparently not necessary for neuronal excitability; (2) Ca^2+^ is essential, but only if present on the outside of the axon, consistent with the old ion-exchange idea referred to before, and, most importantly, (3) excitability is reduced or even lost if, following removal of the axoplasm, the inside of the axon is perfused with either protease containing solutions or, more specifically, drugs interfering with cytoskeletal fibril assembly, growth and/or stability (for more extensive overview see, Drukarch et al., 2018). These conclusions from primarily neurochemical and neurophysiological investigations are largely in line with the observations reported by Le Bihan (2007), who used MRI-technology to study the importance of the interaction between ions and water molecules that enter the neuron during excitation and the subaxolemmal cytoskeleton. In fact, his data led Le Bihan to emphasize that both cytoskeletal integrity and the presence of calcium are necessary for the action potential to occur.

## 4. Conclusion: some final thoughts

As pointed out in the introduction of this paper, it is generally acknowledged that the work of Adrian (1932) and contemporary neurophysiologists has been instrumental in developing our modern theoretical and experimental approaches to investigation, explanation and understanding of the role of action potentials in neuronal physiology and function. Through the clever use of technological innovations in electronic communications technology for the detailed study of the electrical properties and manifestations of the nerve signal, they transformed the ill-defined “propagated disturbance” and “action current” of their predecessors into the well-characterized nerve impulse for their successors to model mathematically and study at the systems, cellular and/or molecular level. Moreover, in emphasizing the importance of the all-or none property of the action potential and using concepts from electronic engineering to speculate about and describe its crucial position and contribution to neuronal and nervous system function, Adrian (1932) paved the path for introduction of the notion of neurons and nervous systems as digital information processors and action potentials as the means by which the highly polarized neuronal cells reproducibly integrate incoming information and communicate its content along axons to their output stations at the axon terminals (Garson, 2003, 2015). However, whilst extremely successful in providing the foundations for large areas of contemporary neuroscience, in particular computational neuroscience and neurophysiology, at the same time the apparent success of the electronics-based framework of neuronal excitability introduced by Adrian (1932) distracted from some inconsistencies in its theoretical foundations and inability to plausibly account for experimental observations that show the nerve impulse to be a multi-physics phenomenon, manifesting itself not only by electrical but also by other co-propagating, non-electrical, signs (Drukarch et al., 2018). As discussed here and elsewhere (Drukarch et al., 2018, 2022; Holland et al., 2019), in our view, it might be (renewed) attention for this latter “blind spot” of the “classical,” i.e., electricity-centered, conception of neuronal excitability that may offer new inroads to study and provide novel insights into the physical nature of the nerve signal thereby opening a window of opportunity to gain a fuller understanding of neuronal physiology and function and the role of action potentials therein. Indeed, this line of reasoning is supported by the growing agreement amongst some groups of bioscientists that our current conception of neuronal excitability is too narrow and should be widened to incorporate all relevant physical, electrical as well as non-electrical, aspects of the nerve impulse into a “fully coupled model” (Mueller and Tyler, 2014; Engelbrecht et al., 2018; Jerusalem et al., 2019). However, hampered not only by technological and conceptual challenges for those working on it (for discussion see, Drukarch et al., 2018, 2022), the debate on the (multi)physics and proper modeling of action potential propagation is also riddled by misunderstandings between those involved (see for instance, Fox, 2018) and, as a consequence, is largely ignored by mainstream neuroscience. This may well be an unfortunate state of affairs, as in recent years it is increasingly recognized that the generally accepted, HH-model based, electrical framework of the action potential, albeit providing much of the foundations of computational neuroscience and (cellular) neurophysiology, cannot be easily integrated with other areas of neurobiological investigation, in particular chemocentric systems (neuro)biology (Almog and Korngreen, 2016). Also, other potential shortcomings of the current approach to investigation, modeling and description of the discharge of action potentials as the prime manifestation of the activity of single neurons operating as optimally integrated individual cellular units have been identified and discussed (see above). Thus, alternative explanations for the rapid and robust transport of information by action potentials along the axonal surface may be sought for. To this end, and based on evidence and ideas gleaned from literature and presented in the previous sections, we propose that treating the nerve signal as a “mesoscopic”-level phenomenon, naturally emerging after appropriate stimulation from the collective material properties of the “axolemma- ectoplasm complex” residing and actively kept in a state near a phase transition under the prevailing environmental conditions surrounding the axonal membrane, will prove to be a fruitful approach to develop a different, more comprehensive and holistic, perspective on the physico-chemical fundaments of neuronal excitability. Indeed, in trying to provoke discussion and provide an initial sketch of what such a novel perspective, based on consecutively interacting volume- and lipid membrane phase transitions, might entail, Wnek (2016) notes that “as the ectoplasm is bonded to the underside of the membrane, there may be a synergistic coupling between radial ion exchange and the proposed signal propagation in the ectoplasm (e.g., a pulse along the nanofiber matrix) and Heimburg’s “pulse along the membrane” model.” Intriguingly, albeit in obvious need of further theoretical exploration and experimental verification, Wnek’s hypothesis aligns well with novel ideas from others who, although pursued from a different theoretical and experimental angle, put forward that during action potential discharge “filaments fire before membrane,” thereby arguing against commonly held belief (Ghosh et al., 2022). Furthermore, in this context it is also of interest that recent data show activity- and/or synchrony-dependent regulation of axonal diameter and conduction velocity or amplitude of action potentials, respectively (Chereau et al., 2017; Zbili et al., 2020), in which axonal swelling is linked to changes in axonal circumferential contractility and cytoskeleton rearrangements (Costa et al., 2018; Yehuda et al., 2021). As such, these results point to an intimate and dynamic relationship between regulation of the characteristics of the electrical manifestation of action potential propagation and the mechanics and physical state of the axonal cytoskeleton. Further experiments are required to unravel the physico-chemical details of this interaction.

In the early 1970s Ichii Tasaki, in a paper co-authored by Tasaki and Hallett (1972), analyzed the process of action potential generation and propagation in relation to the problem of energy transduction in nerves. Whilst doing so, they reflected on difficulties inherent to analyses of excitation processes on a molecular basis. More in particular, they wrote that “From a physicochemical and biochemical point of view, nervous tissue is peculiar, difficult material to investigate. The physico-chemical processes underlying production of an action potential occur within an extremely thin membrane structure and progress at a disturbingly high rate. Most of the chemists’ standard tools are totally inadequate to follow such rapid processes involving such a limited quantity of chemical substance in labile, “living” tissue. Only electronic devices employed by communication engineers have had the sensitivity and the rapidity to respond to the signs of physicochemical events taking place in the nerve membrane. For this reason, “axonology” has developed almost as a branch of applied electronic engineering.” On a positive note, they continued with the observation that in spite of this, “many “axonologists” are keenly aware of the fact that precise measurements of electrical quantities alone do not yield meaningful information about what is happening in the nerve membrane.” Most importantly, from this they concluded that at the time it was already widely recognized that “the goal and the destiny of “axonology” is toward harmony and amalgamation with the branch of science known as molecular biology,” and that for this reason “New instruments and techniques introduced …. to study excitation processes may be regarded as products of a painstaking struggle to achieve this goal.” From our focused analysis of (some of the) developments since then and the current state of affairs concerning elucidation of the mechanism(s) of neuronal excitability, in general, and the action potential phenomenon, in particular, it would seem that this struggle is still ongoing and, in light of the potential importance of its outcome for our understanding of brain and nervous system function, the neurosciences should welcome and be open to different perspectives on modeling and explanatory understanding of the physics of the nerve signal (for concise comparison, see Table 1).

**TABLE 1:** Different perspectives on the physics of the action potential.

Electricity-centered framework	Thermodynamics-centered framework
- Reductionistic/mechanistic approach to modeling and explanatory understanding	- Holistic/non-mechanistic approach to modeling and explanatory understanding
- Focus on electric phenomenology (flow of ionic current)	- Focus on macroscopic thermodynamics (state variables)
- Focus on functional transformations in the properties and activity of microscopic cellular entities (ion channels)	- Focus on structural transformations (phase transitions) in the collective, physico-chemical, properties of polymeric cellular entities (lipid cell membrane, cytoskeletal elements)
- Non-electrical phenomena of action potentials treated as epiphenomena	- Electrical as well as non-electrical phenomena treated as the consequence of a single physical process.
- Propagation is the result of an irreversible, energy-consuming process	- Propagation is the result of a (largely) reversible, energy-conserving process

## Data availability statement

The original contributions presented in this study are included in the article/supplementary material, further inquiries can be directed to the corresponding author.

## Author contributions

Both authors listed have made a substantial, direct, and intellectual contribution to the work, and approved it for publication.
